# 超高效液相色谱-高分辨质谱法测定中华绒螯蟹中游离氨基酸

**DOI:** 10.3724/SP.J.1123.2022.03027

**Published:** 2022-09-08

**Authors:** Ling GAO, Qiang GU, Hong WANG, Xingkong MA, Feng XUE, Xing ZHANG, Jiachun GE, Tao DING, Weijian SHEN

**Affiliations:** 1.南京海关动植物与食品检测中心, 江苏 南京 210019; 1. Animal, Plant and Food Inspection Center, Nanjing Customs, Nanjing 210019, China; 2.张家港海关综合技术中心, 江苏 张家港 215600; 2. Comprehensive Technology Center of Zhangjiagang Customs, Zhangjiagang 215600, China; 3.江苏省淡水水产研究所, 江苏 南京 210017; 3. Freshwater Fisheries Research Institute of Jiangsu Province, Nanjing 210017, China; 4.南京农业大学动物医学院, 江苏 南京 210095; 4. College of Veterinary Medicine, Nanjing Agricultural University, Nanjing 210095, China; 5.南京师范大学食品与制药工程学院, 江苏 南京 210023; 5. School of Food Science and Pharmaceutical Engineering, Nanjing Normal University, Nanjing 210023, China

**Keywords:** 超高效液相色谱-高分辨质谱, 游离氨基酸, 中华绒螯蟹, ultra-high performance liquid chromatography-high resolution mass spectrometry (UHPLC-HRMS), free amino acid, Eriocheir sinensis

## Abstract

游离氨基酸不仅是一类重要的营养物质,更与中华绒螯蟹独特的滋味和香味密切相关。对游离氨基酸含量进行测定,能为中华绒螯蟹产品的品质评价和风味研究提供有价值的信息。该工作通过优选提取溶剂、优化仪器测定参数,最终使用5%(v/v)高氯酸水溶液提取目标物,采用XDB-C_18_色谱柱(100 mm×4.6 mm, 1.7 μm),以0.1%(v/v)甲酸水溶液-乙腈为流动相进行梯度洗脱,在电喷雾正离子电离、选择离子扫描模式下进行检测,外标法定量,建立了超高效液相色谱-高分辨质谱(UHPLC-HRMS)测定中华绒螯蟹中17种游离氨基酸含量的方法。结果表明,17种氨基酸在10.0~200.0 mg/L范围内线性关系良好,相关系数(*R*^2^)≥0.9990,仪器检出限为0.3 mg/L,仪器定量限为1.0 mg/L。在中华绒螯蟹可食部分中按三水平进行加标回收试验,各目标物的回收率在78.4%~105.3%之间。通过对仪器和方法精密度进行评估,发现17种氨基酸测定仪器重复性的RSD≤4.2%,方法重复性试验的RSD≤5.2%,再现性试验的RSD≤11.4%,精密度数据均在合理范围内。利用该研究建立的检测方法对20个中华绒螯蟹样本进行了实际样品检测,总结了17种游离氨基酸在蟹肉、蟹膏、蟹黄中的分布特点,验证了该方法是一种前处理简便、选择性好、准确度高的检测技术。

## Abstract (en)

Eriocheir sinensis is a unique freshwater crab found in China, which is well known for its rich nutrition and sweet and delicious taste. Free amino acids in Eriocheir sinensis are not only important nutrients but also are closely related to their unique taste and aroma. Therefore, the determination of the free amino acid contents in Eriocheir sinensis is of great significance for product quality evaluation, flavor research, authenticity, and origin identification. Herein we proposed an ultra-high performance liquid chromatography-high resolution mass spectrometry (UHPLC-HRMS)-based method for the determination of 17 free amino acids in Eriocheir sinensis. First, 5 g of the Eriocheir sinensis sample was weighed into a 50-mL polypropylene centrifuge tube. Then, 10 mL of extraction solvents was added to the centrifuge tube, and the resultant solution was mixed well using a vortex mixer. We compared a variety of solvents and finally selected 5%(v/v) perchloric acid aqueous solution as the optimum extraction solvent. The supernatant was transferred to another polypropylene centrifuge tube after centrifuging at 8000 r/min for 5 min. The extraction procedure was repeated according to the above-mentioned steps, and the extraction solution was combined with the supernatant. The extracts were then adjusted to pH 6.5 with 1 mol/L potassium hydroxide solution, and were diluted to 50 mL with water. After filtering by both qualitative filter paper and a 0.45-μm polyether sulfone syringe filter, the extracts were determined by UHPLC-HRMS. We compared three types of mobile phases and chose 0.1%(v/v) formic acid aqueous solution mixed with acetonitrile as the optimum one. Precise parent ion and ion source parameters were also optimized. The 17 analytes, viz. aspartic acid, threonine, serine, glutamic acid, proline, cystine, valine, methionine, isoleucine, leucine, tyrosine, phenylalanine, lysine, arginine, glycine, alanine, and histidine, were separated on an XDB-C_18_ column (100 mm×4.6 mm, 1.7 μm) with gradient elution. The amino acids were then detected by HRMS in electrospray ionization and selected ion monitoring modes, and the analytes were quantified using external standards. The instrumental detection limit (IDL) and the instrumental quantification limit (IQL) were 0.3 mg/L and 1.0 mg/L, respectively. The linear correlation coefficients were all above 0.9990 in the concentration range of 10.0-200.0 mg/kg. Three levels of free amino acid standards were spiked into the edible parts of Eriocheir sinensis. The recoveries of the amino acids were between 78.4% and 105.3%. The intra-sample, intra-day, and inter-day repeatabilities were below 4.2%, 5.2%, and 11.4%, respectively, which were within reasonable ranges. Twenty samples of Eriocheir sinensis were tested using the proposed method. Thus, in this study, we developed an alternative method for the determination of free amino acids in Eriocheir sinensis with simple pretreatment, good selectivity, and high accuracy.

中华绒螯蟹俗称大闸蟹,是一种我国所特有的淡水蟹,其以味甜鲜美、富有营养而久负盛名。中华绒螯蟹中的游离氨基酸不仅是一类重要的营养物质,而且与蟹独特的滋味和香味密切相关^[1]^。如谷氨酸、天门冬氨酸等氨基酸具有鲜味的特征,甘氨酸、色氨酸、丙氨酸等氨基酸甜味明显,而亮氨酸、异亮氨酸等氨基酸则有明显的苦味^[2]^。由于养殖环境、水质、所投喂饲料等因素的差异,不同产地中华绒螯蟹中的游离氨基酸总量、组成有所差别,使这些产品之间的味道呈现出微妙的差异^[3]^,从而极大地影响了产品的价格。因此,对中华绒螯蟹中游离氨基酸组成和含量进行测定,对其产品品质评价、风味研究、真伪和产地鉴别等都有重要意义。

食品中氨基酸的含量大多采用液相色谱法测定^[4⇓⇓⇓⇓⇓-10]^,样品中氨基酸经提取、净化后,使用衍生化试剂进行柱前衍生,液相色谱分离后采用紫外或二极管阵列检测器测定。也有文献采用气相色谱法^[11]^、气相色谱-质谱联用法^[12,13]^、离子交换色谱法^[14,15]^、毛细管电泳法^[16]^进行测定,但也均需对氨基酸进行衍生,而衍生化反应操作较为繁琐,且存在衍生物不稳定的缺点^[5]^。氨基酸自动分析仪^[1]^、电位传感法^[17]^操作简便且无需进行衍生化反应,适合快速筛查和现场检测工作,但这些方法很难实现不同氨基酸之间的基线分离,抗干扰能力弱,不适合检测基质复杂的大闸蟹样品。质谱法同样无需衍生化反应,又兼具灵敏度高、选择性好的特点,已经被成功用于茶叶、芒果、婴儿食品中游离氨基酸的测定^[18⇓⇓-21]^。而四极杆-静电场轨道阱高分辨质谱作为新一代的高分辨质谱技术,将四极杆的高离子选择性和静电场轨道阱高分辨扫描技术有机结合,不仅具有普通串联质谱较高灵敏度和选择性的优点,更进一步地提高了采集质量精度、灵敏度等。同时,高分辨质谱无需对目标物逐个优化子离子及相关参数,仅通过全扫描和数据依赖性二级扫描模式,一次进样分析即可同时完成一级和二级质谱扫描,既能得到一级的精确质量数,也能获得二级的特征碎片离子等信息,弥补了传统三重四极杆质谱检测化合物数量有限、假阳性判断能力不足的缺陷。袁光蔚等^[22]^采用超高效液相色谱-四极杆-静电场轨道阱高分辨质谱(UHPLC-HRMS)建立了水果中18种游离氨基酸的测定方法,王忠合等^[23]^采用亲水作用色谱-飞行时间质谱测定了单丛乌龙茶中的游离氨基酸,都证明了高分辨质谱可有效排除复杂基质的干扰,从而提高测定准确度。目前,尚未见利用四极杆-静电场轨道阱高分辨质谱测定中华绒螯蟹中游离氨基酸的报道。

本工作采用UHPLC-HRMS技术对中华绒螯蟹中17种游离氨基酸进行了快速测定,并对20份取自江苏地区的中华绒螯蟹样品进行了实际样本检测和验证。此项工作将为中华绒螯蟹的品质评价和风味研究提供有价值的技术信息。

## 1 实验部分

### 1.1 仪器、试剂与材料

超高效液相色谱-四极杆-静电场轨道阱高分辨质谱联用仪(美国Thermo Fisher公司); XP205电子天平(瑞士Mettler Toledo公司); Lab Dancer涡旋振荡仪(德国IKA公司); X4R高速离心机(美国Thermo Fisher公司); S220-K-CN pH计(瑞士Mettler Toledo公司)。

17种氨基酸标准品(纯度>99%)购自北京振翔科技有限公司,详细信息见表1;氢氧化钾(分析纯)、高氯酸(分析纯)、定性滤纸购自国药集团化学试剂有限公司;乙腈(色谱纯)、甲酸(色谱纯)、甲醇(色谱纯)、0.45 μm水相微孔滤膜购自上海安谱实验科技股份有限公司。

**表 1:** 17种氨基酸的CAS号、精确质荷比和保留时间

No.	Amino acid	CAS number	Accurate m/z^*^	Retention time/min
1	Asp (天门冬氨酸)	56-84-8	134.0434	1.88
2	Thr (苏氨酸)	72-19-5	120.0645	5.92
3	Ser (丝氨酸)	302-84-1	106.0491	1.85
4	Glu (谷氨酸)	56-86-0	148.0412	1.90
5	Pro (脯氨酸)	29795-82-2	116.0697	1.96
6	Cys (胱氨酸)	56-89-3	241.0286	1.82
7	Val (缬氨酸)	7004/3/7	118.0853	2.26
8	Met (甲硫氨酸)	63-68-3	150.0568	2.53
9	Ile (异亮氨酸)	131598-62-4	132.1007	3.29
10	Leu (亮氨酸)	61-90-5	132.1007	3.54
11	Tyr (酪氨酸)	60-18-4	182.0794	3.36
12	Phe (苯丙氨酸)	63-91-2	166.0845	5.92
13	Lys (赖氨酸)	56-87-1	147.1113	1.71
14	Arg (精氨酸)	74-79-3	175.1171	1.74
15	Gly (甘氨酸)	56-40-6	76.0390	1.83
16	Ala (丙氨酸)	56-41-7	90.0544	1.87
17	His (组氨酸)	71-00-1	156.0750	1.73

* [M+H]^+^, quantitative ion.

分别称取17种氨基酸标准品各10 mg,混合后用水溶解并定容至100 mL,此混合标准溶液中各氨基酸质量浓度均为100 mg/L。吸取上述混合标准溶液100、200、500、1000、2000 μL于10 mL容量瓶中,分别加水定容,配制成氨基酸混合标准工作系列溶液,质量浓度分别为1、2、5、10、20 mg/L。

### 1.2 样品前处理

称取5.00 g试样于50 mL聚丙烯离心管中,加入10 mL 5%(v/v)高氯酸水溶液,用涡旋振荡器充分混匀,再以8000 r/min离心5 min,取上清液于另一聚丙烯离心管中。按上述步骤用10 mL 5%(v/v)高氯酸溶液重复提取一次,合并提取液。用1 mol/L氢氧化钾溶液调节提取液pH至6.5,转移至50 mL容量瓶中,用水定容至刻度线。定性滤纸过滤后,用0.45 μm水相微孔滤膜过滤,供UHPLC-HRMS测定。对于中华绒螯蟹中含量较高的氨基酸,需进一步稀释,使其最终浓度在标准曲线范围之内。

### 1.3 分析条件

#### 1.3.1 色谱条件

色谱柱:XDB-C_18_色谱柱(100 mm×4.6 mm, 1.7 μm);柱温:25 ℃;流动相:A为0.1%甲酸水溶液,B为乙腈。梯度洗脱程序:0~0.8 min, 2%B; 0.8~3.0 min, 2%B~24%B; 3.0~4.0 min, 24%B; 4.0~6.0 min, 24%B~95%B; 6.0~9.0 min, 95%B; 9.0~9.5 min, 95%B~2%B; 9.5~12.0 min, 2%B。流速:0.6 mL/min;进样量:5 μL。

#### 1.3.2 质谱条件

离子源:电喷雾离子源(ESI),正离子模式;毛细管温度:350 ℃;鞘气(N_2_)流速50 L/min;辅助气(N_2_)流速6 L/min;吹扫气(N_2_)流速:3 L/min;喷雾电压:3 kV;透镜电压:50 V;一级质谱扫描分辨率(*R*): 70000;自动增益控制(AGC): 2×10^5^;最大驻留时间:100 ms;分离窗口:*m/z* 2.0。17种氨基酸标准品的CAS号、监测离子、保留时间等见表1。

## 2 结果与讨论

### 2.1 提取溶剂的选择

采用UHPLC-HRMS对中华绒螯蟹中游离氨基酸进行测定的方法具有选择性好、抗干扰能力特别强的优点。其无需彻底的液相色谱基线分离和基质净化,仅需提取、离心、过滤等简便操作,就能实现目标物的准确定性和定量测定。然而,即便如此,提取步骤依然是决定检测准确性的重要环节,有必要对影响提取效率的最重要因素——提取溶剂进行优选。戴明等^[18]^在测定茶叶中游离氨基酸时,对比了12种提取溶剂的提取效果后,发现20%(v/v)甲醇水溶液的提取效率最佳。同样是测定茶叶中的游离氨基酸,杜颖颖等^[19]^发现在水中加入少量的甲酸后,能大幅提升提取效率,甚至高于乙醇或甲醇水溶液。臧彬如等^[24]^使用水提取,测定了鹿茸中16种氨基酸,平均加标回收率达到了98.9%。这些工作为本方法的开发带来了启发,然而,中华绒螯蟹具有高蛋白、高油脂的特点,在提取环节中需要尽量排除这些物质对后续测定的干扰。一些酸性沉淀剂,如高氯酸、三氯乙酸等具有沉淀蛋白质的作用,也常被用于中华绒螯蟹氨基酸、核苷酸等成分的提取^[3,25,26]^。

本工作首先对比了20%(v/v)甲醇水溶液、0.2%(v/v)甲酸水溶液、5%(v/v)高氯酸水溶液等3种提取试剂的提取效率。当使用20%(v/v)甲醇水溶液、0.2%(v/v)甲酸水溶液作为提取溶剂时,使用氢氧化钾溶液或甲酸调节合并的提取液pH至6.5,其他步骤不变。我们在中华绒螯蟹样本中添加了一定量的氨基酸标准品,确保每一种氨基酸都有检出。分别采用3种提取溶剂对该加标样本进行提取后测定,通过对比每种氨基酸测得含量的大小,来评估3种提取溶剂的提取效率。如图1所示,采用20%(v/v)甲醇水溶液提取时,仅异亮氨酸、酪氨酸的提取效率较高,这可能与这两种氨基酸具有相对较长的疏水基团有关。但该溶剂对其他游离氨基酸的提取效果并不理想,特别是脯氨酸、缬氨酸、甘氨酸的提取效率明显不如另两种溶剂。0.2%(v/v)甲酸水溶液在提取脯氨酸时效率最高,但对其他氨基酸的提取效率均不如5%(v/v)高氯酸水溶液,特别是对天门冬氨酸、谷氨酸的提取效率较低,可能是由于这两种氨基酸的等电点在2~4左右,与甲酸水溶液的pH值非常接近,导致这两种氨基酸在该提取溶剂中的溶解度不高。因此,与20%(v/v)甲醇水溶液和0.2%(v/v)甲酸水溶液相比,5%(v/v)高氯酸水溶液的整体提取效率较高。

**图 1:**
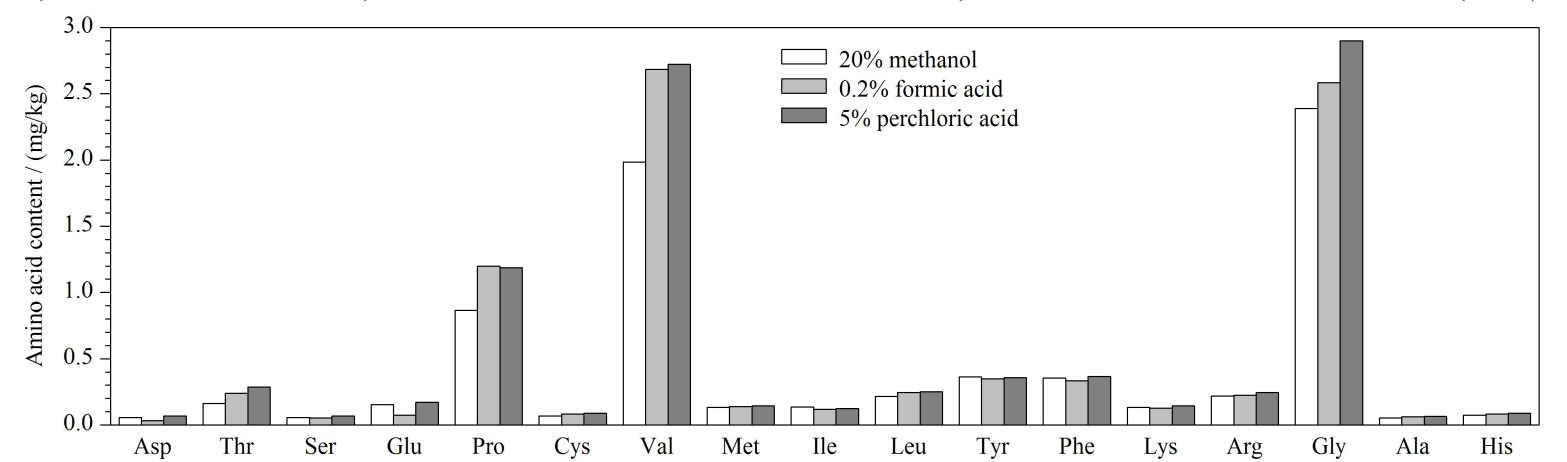
采用3种不同提取溶剂时的氨基酸测得值

此外,本工作对比了1%、2%、5%、10%(v/v)4种不同含量高氯酸水溶液的提取效率。如图2所示,当高氯酸含量从1%提升到5%时,大部分氨基酸的提取效率随高氯酸含量的升高而提升,特别是天门冬氨酸、丝氨酸、缬氨酸、甘氨酸等的提升非常显著。而当高氯酸含量从5%提升到10%时,脯氨酸提取效率略有下降,甲硫氨酸、缬氨酸则略有上升,但大多数氨基酸的提取效率基本维持不变。因此,5%和10%高氯酸水溶液均能获得较理想的提取效率。从环保和操作安全的角度考虑,本工作选择5%(v/v)高氯酸水溶液作为提取溶剂。

**图 2:**
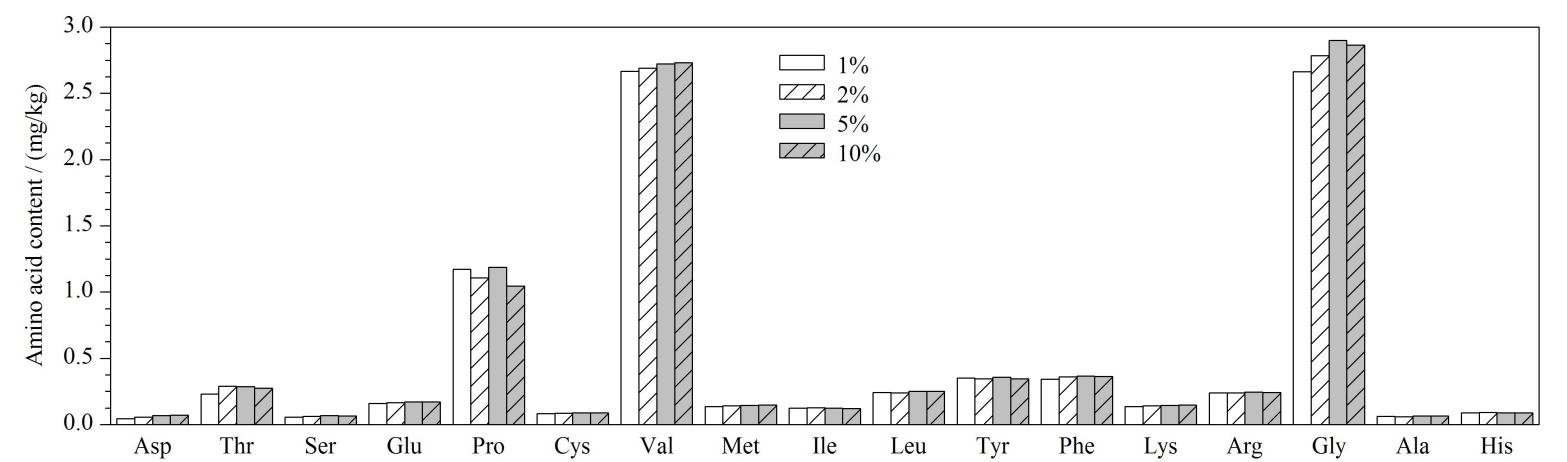
采用4种含量的高氯酸水溶液提取的氨基酸含量

### 2.2 仪器条件优化

在氨基酸的液相色谱分离中,有采用亲水作用色谱(hydrophilic-interaction chromatography, HILIC)的^[27]^,也有使用C_8_或C_18_反相液相色谱的,都成功实现了氨基酸的分离^[18]^,本工作选用食品分析实验室最为常用的C_18_色谱柱。比较了水-乙腈、0.1%(v/v)甲酸水溶液-乙腈、5 mmol/L乙酸铵水溶液-乙腈作为流动相时17种氨基酸的分离和色谱峰情况。在ESI^+^模式下,流动相的离子强度大小会直接影响氨基酸的离子化效率,进而决定质谱响应强度。结果表明,使用水-乙腈作为流动相时,氨基酸的响应较低。与5 mmol/L乙酸铵水溶液-乙腈相比,使用0.1%(v/v)甲酸水溶液-乙腈作为流动相时,氨基酸的峰形更好、响应更强。因此,最终确定使用0.1%(v/v)甲酸水溶液-乙腈作为流动相。

在质谱参数方面,本工作采用流动注射分析的方式将17种氨基酸标准溶液分别注入质谱仪,通过全扫描得到精确母离子[M+H]^+^的质荷比,并同时优化了相关参数,优化条件下17种氨基酸标准溶液的色谱图见图3。

**图 3:**
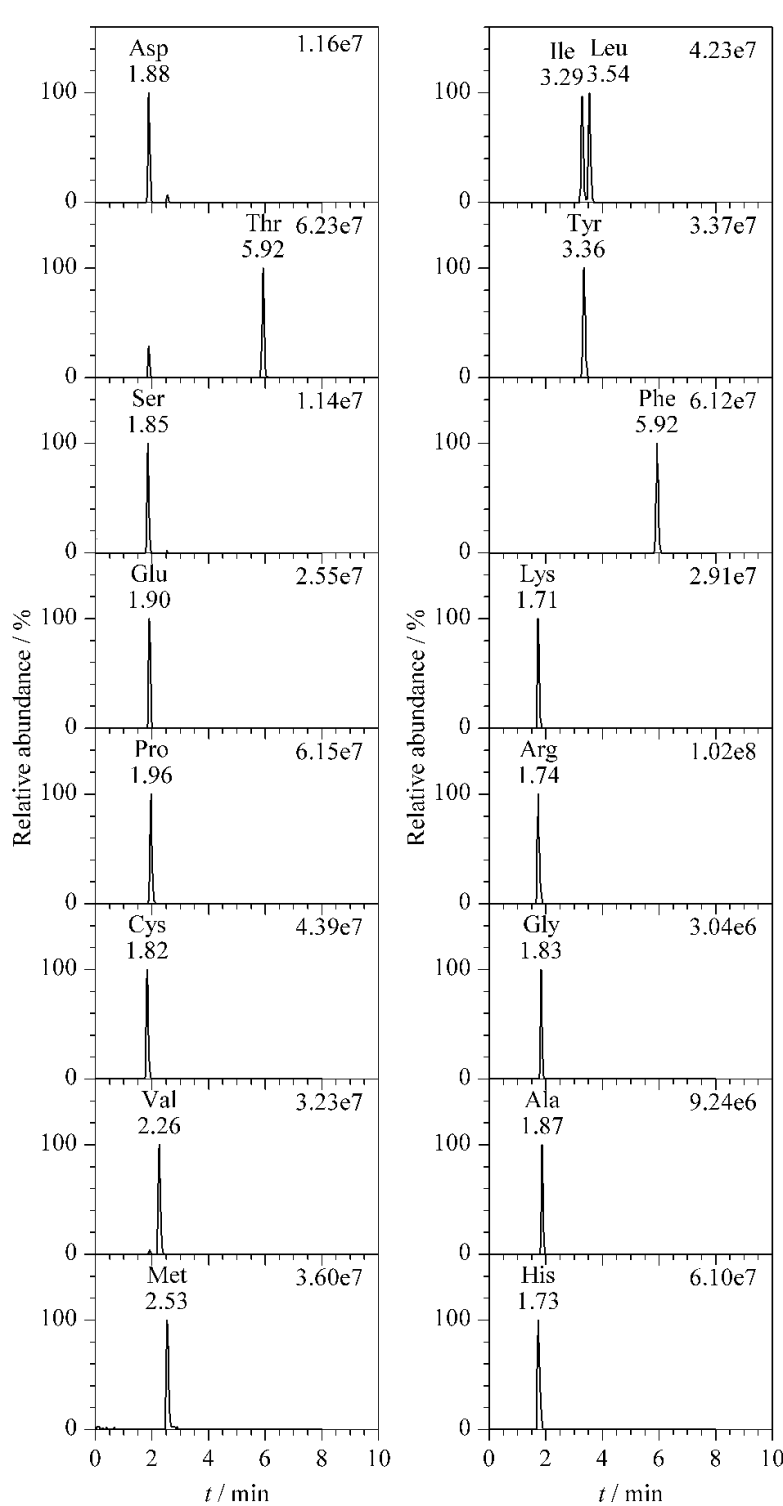
17种氨基酸标准溶液(10 mg/L)的色谱图

### 2.3 线性范围、仪器检出限和定量限

分别取适量17种氨基酸标准溶液,用超纯水稀释,按1.3.1节及1.3.2节色谱和质谱条件测定,以各标准物质的质量浓度(*x*, mg/L)为横坐标,峰面积(*y*)为纵坐标绘制标准曲线,计算回归方程。

按信噪比(*S/N*)=3计算仪器检出限(IDL), *S/N*=10计算仪器定量限(IQL)。17种氨基酸的线性范围为10.0~200.0 mg/L,在该范围内相关系数(*R*^2^)均≥0.9990,线性良好。17种氨基酸的线性范围、回归方程、*R*^2^、IDL、IQL见表2。

**表 2:** 17种氨基酸的线性回归方程、相关系数、仪器检出限 和仪器定量限

No.	Amino acid	Regression equation	R^2^	IDL/ (mg/L)	IQL/ (mg/L)
1	Asp	y=127.085x+1.04	0.9996	0.3	1.0
2	Thr	y=361.861x+2.55	0.9994	0.3	1.0
3	Ser	y=132.796x+1.04	0.9999	0.3	1.0
4	Glu	y=263.056x+4.61	0.9997	0.3	1.0
5	Pro	y=63.982x+7.41	0.9990	0.3	1.0
6	Cys	y=616.690x+5.43	0.9994	0.3	1.0
7	Val	y=454.423x+6.28	0.9996	0.3	1.0
8	Met	y=437.886x+1.26	0.9992	0.3	1.0
9	Ile	y=223.997x+4.16	0.9990	0.3	1.0
10	Leu	y=180.349x+2.57	0.9996	0.3	1.0
11	Tyr	y=777.309x+2.38	0.9998	0.3	1.0
12	Phe	y=185.718x+7.09	0.9992	0.3	1.0
13	Lys	y=637.939x+3.26	0.9994	0.3	1.0
14	Arg	y=270.982x+2.34	0.9996	0.3	1.0
15	Gly	y=474.529x+4.57	0.9991	0.3	1.0
16	Ala	y=307.261x+3.88	0.9992	0.3	1.0
17	His	y=435.605x+7.12	0.9991	0.3	1.0

IDL: instrumental detection limit; IQL: instrumental quantitation limit. *y*: peak area of the quantitative ion; *x*: mass concentration, mg/L.

### 2.4 精密度和回收率

取一中华绒螯蟹样本的可食部分粉碎混匀,在其中分别添加3个浓度水平的氨基酸标准品。一般来说,加标回收试验需要从定量限浓度开始,然而,中华绒螯蟹中游离氨基酸的本底值较高,需要在更高的浓度下加标,以更好地反映实际检测时的方法准确度。在本工作中,脯氨酸、缬氨酸、甘氨酸的加标水平为500、1000、2000 mg/kg,其他氨基酸为50、100、200 mg/kg。

#### 2.4.1 精密度

中华绒螯蟹中17种游离氨基酸含量的UHPLC-HRMS测定方法的精密度评估由仪器重复性、方法重复性和再现性试验组成,采用上述制备的最低浓度加标水平的样本进行。仪器重复性试验:使用同样的仪器条件对同一份样品提取液连续测定6次完成,计算测定结果的平均值及相对标准偏差(RSD);方法重复性试验:由同一操作者在同一天内连续测定6个同一加标水平的样本;再现性试验:由不同的操作者在连续5天中分别测定5个同一加标水平的样本。如表3所示,17种氨基酸仪器重复性试验的RSD≤4.2%,方法重复性试验的RSD≤11.4%,再现性试验的RSD≤13.9%。其中,Asp、Ser、Ile、Leu、Ala、His等6种游离氨基酸的精密度数据符合食品理化检测要求。而其余11种游离氨基酸的方法重复性试验的RSD为4.6%~11.4%,超出了GB/T 27404-2008《实验室质量控制规范 食品理化检测》附录F3中规定的范围,因此,如需将这11个指标用于相关研究工作,需要评估精密度数据对研究结果的影响。

**表 3:** UHPLC-HRMS 方法的精密度

Amino acid	Contents/(mg/kg) (RSDs/%)		
	Intra-sample (n=6)	Intra-day (n=6)	Inter-day (n=5)
Asp	72.0 (2.3)	68.2 (5.2)	71.5 (8.5)
Thr	281.9 (1.8)	287.5 (3.2)	316.0 (4.1)
Ser	64.1 (3.0)	67.3 (3.5)	66.8 (11.7)
Glu	153.6 (3.2)	169.9 (6.4)	178.5 (3.3)
Pro	1061.0 (2.2)	1147.2 (2.6)	1125.5 (1.9)
Cys	88.6 (1.7)	87.3 (2.4)	89.3 (3.0)
Val	2181.4 (1.3)	2722.2 (1.7)	2683.6 (2.1)
Met	142.7 (3.0)	145.4 (2.9)	144.8 (3.3)
Ile	120.2 (4.2)	125.3 (3.5)	129.9 (7.6)
Leu	232.8 (3.1)	250.1 (3.3)	225.6 (9.8)
Tyr	341.3 (2.6)	356.0 (3.4)	347.4 (4.1)
Phe	356.6 (2.5)	366.6 (3.0)	369.1 (3.8)
Lys	160.0 (3.7)	144.7 (2.4)	157.5 (1.9)
Arg	252.1 (3.0)	243.8 (2.7)	247.0 (3.7)
Gly	2630.4 (2.5)	2898.4 (3.2)	2737.1 (1.7)
Ala	62.3 (1.6)	65.7 (4.2)	66.8 (5.5)
His	87.5 (3.4)	89.2 (2.9)	83.1 (5.0)

#### 2.4.2 回收率

加标回收率试验是采用3个加标水平的中华绒螯蟹样品进行的,每个样品重复测定6次,每个指标的加标水平及相应回收率数据如表4所示,17种氨基酸的加标回收率在78.4%~105.3%之间。

**表 4:** 样品中游离氨基酸的加标回收率(*n*=6)

Amino acid	Background/ (mg/kg)	Spiked/ (mg/kg)	Recovery/ %	RSD/ %
Asp	22.2	50	92.4	4.6
		100	89.6	3.7
		200	86.7	5.2
Thr	239.5	50	84.7	5.3
		100	82.6	4.3
		200	84.8	5.9
Ser	25.8	50	82.6	3.7
		100	79.3	5.2
		200	84.0	2.6
Glu	109.6	50	88.1	5.9
		100	92.5	6.0
		200	95.1	4.3
Pro	598.5	500	92.5	4.5
		1000	88.3	6.8
		2000	90.1	5.7
Cys	42.0	50	93.3	6.1
		100	95.7	3.0
		200	98.4	1.5
Val	1723.4	500	91.6	5.1
		1000	85.5	4.7
		2000	87.3	5.3
Met	96.4	50	92.7	6.2
		100	89.0	4.6
		200	95.2	2.8
Ile	82.8	50	84.6	4.0
		100	86.3	5.8
		200	83.2	3.5
Leu	208.6	50	83.3	2.6
		100	82.1	4.4
		200	80.9	2.9
Tyr	299.0	50	84.7	6.9
		100	78.4	5.7
		200	80.7	3.1
Phe	314.6	50	84.1	4.5
		100	89.0	4.9
		200	84.6	5.1
Lys	115.7	50	88.5	5.5
		100	84.9	6.0
		200	82.2	8.5
Arg	209.8	50	84.6	5.0
		100	97.1	4.1
		200	93.7	5.4
Gly	2188.9	500	88.3	4.3
		1000	87.2	3.7
		2000	85.0	4.5
Ala	18.2	50	90.1	4.4
		100	82.7	9.7
		200	105.3	7.1
His	42.7	50	84.6	3.8
		100	86.9	5.8
		200	92.5	4.6

### 2.5 实际样品检测

取养殖的中华绒螯蟹样品20份(江苏省宿迁市,其中公蟹和母蟹各10份),每份样品分别剥离出蟹肉(体肉、爪肉、螯肉)、母蟹蟹黄(母蟹肝胰腺和性腺)或公蟹蟹膏(公蟹肝胰腺和性腺),分别混匀装袋。使用本工作建立的检测方法对上述各样品中游离氨基酸含量进行测定,结果见表5。从表5中可以看出,在游离氨基酸总量上,公蟹肉比公蟹膏的含量高,母蟹肉也比母蟹黄的含量高,这与郭宏慧等^[26]^的研究结果一致。此外,每一种游离氨基酸在蟹肉与蟹膏/蟹黄中的分布又都不一样,如甘氨酸在蟹肉中的含量要比蟹膏和蟹黄中的含量高得多,由于甘氨酸是一种甜味氨基酸,这是蟹肉口味要更加甘甜的原因之一。而很多游离氨基酸在蟹膏和蟹黄中的含量却比蟹肉更高,如酪氨酸、苯丙氨酸、苏氨酸、亮氨酸、异亮氨酸等,这些组成特点也赋予了蟹膏和蟹黄更为立体的口味。

**表 5:** 中华绒螯蟹不同部位游离氨基酸含量(*n*=10)

Amino acid	Male crab meat	Crab paste	Female crab meat	Crab yolk
Asp	4.0±3.5	56.8±22	2.6±0.8	7.6±5.5
Thr	214.6±46.5	385.2±162.5	262.5±40.4	446.8±238.9
Ser	17.2±8.0	19.3±6.7	14.7±5.1	20.6±11.0
Glu	91.5±15.1	186.4±51.3	80.5±9.1	184.6±83.8
Pro	896.1±287.1	484.1±129.0	1096.0±246.2	691.9±157.6
Cys	40.9±13.7	27.7±2.8	55.0±14.8	37.9±27.6
Val	2048.4±121.7	2255.3±100.0	1692.6±194.6	2071.7±209.3
Met	95.0±24.4	115.7±48.6	146.2±42.9	163.8±94.2
Ile	95.7±12.2	184.3±39.2	117.7±28.1	216.0±62.0
Leu	163.1±33.3	313.8±50.7	200.4±38.8	367.9±75.6
Tyr	269.5±40.3	432.4±130.7	334.0±72.7	539.1±277.0
Phe	267.0±60.8	491.6±211.9	336.2±59.4	573.6±314.4
Lys	85.3±15.5	129.3±43.7	94.8±16.5	154.8±70.1
Arg	312.6±62.1	124.0±21.4	313.1±46.4	157.2±49.5
Gly	3599.2±1330.4	851.1±205.8	4752.4±1193.3	1116.9±67.4
Ala	15.3±5.1	10.3±1.0	20.5±5.5	14.2±10.3
His	28.8±8.0	51.4±1.2	30.3±10.3	48.9±7.4
Total	8244.3±1840.8	6118.8±940.7	9549.6±1981.0	6813.6±1809.1

## 3 结论

本文建立了UHPLC-HRMS检测中华绒螯蟹中17种氨基酸的分析方法,该方法前处理简便,选择性和抗干扰能力特别强,为中华绒螯蟹中游离氨基酸测定提供了一种更为准确的检测技术。
